# Polo-like kinase 4 inhibitor CFI-400945 inhibits carotid arterial neointima formation but increases atherosclerosis

**DOI:** 10.1038/s41420-023-01305-4

**Published:** 2023-02-07

**Authors:** Jiaxing Sun, Darrell Belke, Yu Gui, Yong-Xiang Chen, Shenghua Zhou, Xi-Long Zheng

**Affiliations:** 1 Departments of Biochemistry and Molecular Biology and Physiology and Pharmacology, Cumming School of Medicine, University of Calgary, Calgary, AB Canada; 2 Department of Cardiology, the Second Xiangya Hospital of Central South University, Changsha, China; 3 Department of Cardiac Sciences, Cumming School of Medicine, University of Calgary, Calgary, AB Canada

**Keywords:** Apoptosis, Atherosclerosis

## Abstract

Neointima lesion and atherosclerosis are proliferative vascular diseases associated with deregulated proliferation of vascular smooth muscle cells (SMCs). CFI-400945 is a novel, highly effective anticancer drug that inhibits polo-like kinase 4 (PLK4) and targets mitosis. In this study, we aim to investigate how CFI-400945 affects the development of proliferative vascular diseases. In C57BL/6 mice, neointima formation was generated by complete carotid ligation. In apolipoprotein E knockout (ApoE^−/−^) mice fed a high-fat diet, atherosclerosis was induced by partial carotid ligation. CFI-400945 was directly applied to carotid arteries via a perivascular collar. Our results showed that CFI-400945 drastically inhibited neointima formation but significantly accelerated atherosclerosis. In vitro studies showed that CFI-400945 treatment induced SMC polyploidization and arrested cells in the G2/M phase. CFI-400945 treatment upregulated p53 and p27 expression but decreased p21 and cyclin B1 expression. CFI-400945 also induced SMC apoptosis, which was inhibited by hydroxyurea, a DNA synthesis inhibitor that inhibits polyploidization. Furthermore, CFI-400945 caused supernumerary centrosomes, leading to mitotic failure, resulting in polyploidization. In conclusion, CFI-400945 prevents carotid arterial neointima formation in C57BL/6 mice but accelerates atherosclerosis in ApoE^−/−^ mice, likely through mitotic arrest and subsequent induction of polyploidization and apoptosis.

## Introduction

Proliferative vascular diseases, including neointima formation, restenosis after an angioplasty procedure and atherosclerosis, are closely associated with abnormal proliferation of vascular smooth muscle cells (SMCs). Neointima lesion is considered to be an early stage of atherosclerosis [1–4]. Atherosclerosis is the primary etiology of cardiovascular diseases, which remain the leading cause of morbidity and mortality accounting for approximately 40% of deaths worldwide [5]. Thus, many strategies have been developed to target SMC proliferation. For example, several small molecule drugs, including rapamycin, paclitaxel, ceramide, doxorubicin, mRNA antagonists, and even siRNAs, have been tested for their anti-proliferative potential [6]. Still, none of them are satisfactory due to their limited therapeutic efficacies. Therefore, it is crucial to develop new, effective approaches or drugs for the treatment of proliferative vascular diseases.

CFI-400945, a novel and highly selective polo-like kinase 4 (PLK4) inhibitor, causes dysregulation of centriole duplication, leading to defective mitosis, decreased proliferation, and cell death in cancer cells [7]. It was first discovered in the lab of Dr. Henry, along with several other novel PLK4 inhibitors [8]. After a series of optimizations, Compound 48, which was named CFI-400945, was found to have superior potency and pharmacokinetic properties in mouse models of tumor growth. The first-in-human phase 1 trial has already established the safety and tolerability of CFI-400945 in patients with advanced solid tumors (NCT01954316) [9]. Several other clinical trials have been registered for treating various cancers, such as breast cancer (NCT03624543), acute myeloid leukemia (NCT04730258), and prostate cancer (NCT03385655). Given the unique mechanism underlying CFI-400945 effects on cancer growth, our thought was that CFI-400945 may effectively treat proliferative vascular diseases.

Notably, several studies showed that CFI-400945 causes polyploidy [10–13], a cell state containing more than two sets of homologous chromosomes (>4N DNA). Polyploidy or aneuploidy is the primary mechanism for tumorigenesis [14, 15]; however, CFI-400945 effects are nuanced in that it induces polyploidy but inhibits cancer growth. Some studies reported that CFI-400945 inhibits cancer growth through its induction of apoptotic death [10, 12, 13], and others proposed that senescence induced by CFI-400945 plays a pivotal role [16, 17]. Interestingly, vascular SMCs undergo polyploidization in the aorta of aged or hypertensive animals and humans, which has been well documented [18–20]. Increasing evidence suggests that SMC polyploidization is associated with SMC senescence and apoptosis [21–23] and the pathogenesis of vascular diseases [19]. Thus, it is an intriguing question how CFI-400945 affects SMC ploidy and the progression of proliferative vascular diseases.

In the current study, we found that CFI-400945 drastically inhibited the development of neointima lesion in mouse carotid arteries resulting from complete carotid ligation. Surprisingly, CFI-400945 accelerated atherosclerosis in carotid arteries induced by partial carotid ligation in apolipoprotein E deficient (ApoE^−/−^) mice fed a high-fat diet. Mechanistically, our in vitro studies showed that CFI-400945 arrested SMCs in the G2/M phase and induced polyploidization and subsequent apoptosis.

## Results

### CFI-400945 inhibits neointima formation induced by complete ligation of mouse carotid arteries

**Fig. 1:**
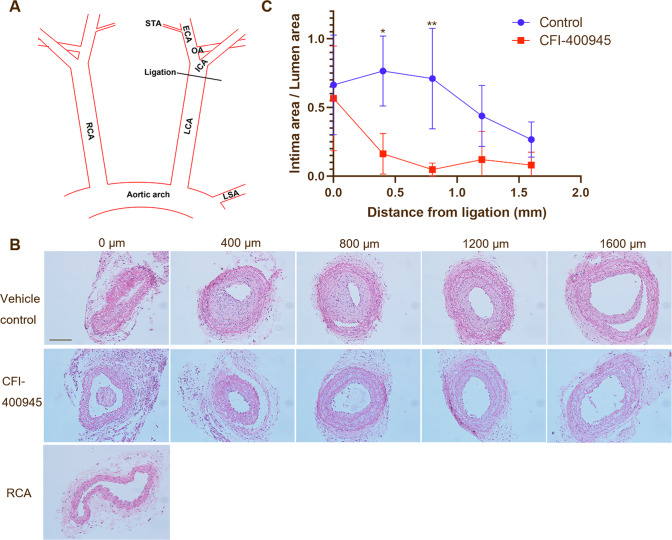
CFI-400945 inhibits neointima formation induced by complete carotid ligation. C57BL/6 J mice received complete ligation of LCA as described in the Methods. CFI-400945 (10 μM) or the same volume of DMSO in the hydrogel was applied onto the ligation site. After three weeks, mice were sacrificed and carotid arteries harvested for analysis. **A** Schematic presentation of complete carotid ligation of LCA, as detailed in the Methods. ECA: external carotid artery; ICA: internal carotid artery; OA: occipital artery. STA: superior thyroid artery. **B** Representative tissue sections (5 µm thickness) from 2 mm proximal to the ligated site were stained with hematoxylin and eosin (HE) for morphometric analyses. Neointima lesions at day 21 after ligation with the vehicle or the CFI-400945 treatment were compared. Scale bar = 100 µm. **C** Cumulative data showing the ratio of intima to lumen area. The data were analyzed with multiple Student’s *t*-tests (*n* = 4). *p < 0.05, **p < 0.01.

To examine if CFI-400945 affects proliferative vascular diseases, we first established the neointima formation model by complete ligation of mouse carotid arteries, as we previously reported [23]. A schematic presentation of the complete carotid ligation of the LCA is shown in Fig. 1A. C57BL/6J mice receiving LCA ligation were divided into two groups treated with vehicle or CFI-400945 as described in the Methods. Our results showed that after 21 days, CFI-400945 drastically inhibited neointima formation (Fig. 1B). The CFI-400945-treated group displayed a significantly lower ratio of intima area to lumen area compared with that in the vehicle control group (Fig. 1C).

### CFI-400945 accelerates atherosclerosis induced by partial ligation of the carotid artery in ApoE^−/−^ mice

**Fig. 2:**
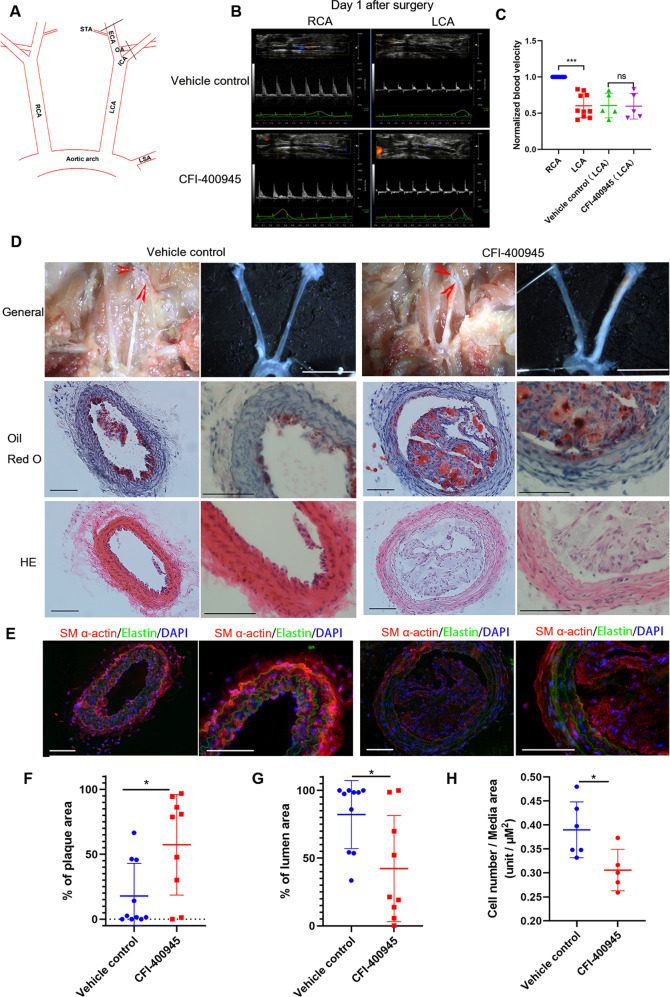
CFI-400945 accelerates atherosclerosis in carotid arteries of ApoE^−/−^ mice induced by partial carotid ligation. **A** Schematic presentation of partial carotid ligation of LCA, as detailed in the Methods. **B** Representative pictures of the ultrasound showing flow velocity profiles revealing that partial ligation reduced blood flow but was not completely blocked after LCA partial ligation both in the vehicle control group and CFI-400945 group on day 1. **C** Cumulative data showing the blood velocity on day 1 after surgery. **D** After 18 days, mice were sacrificed for analyses of carotid atherosclerosis. Upper panels: Representative photos of general carotid artery isolation. Scale bar = 1 cm. Middle panels: Representative tissue sections (10 µm thick) of Oil Red O staining. Scale bar = 100 µm. Lower panels: Representative tissue sections (10 µm thick) of HE staining. Scale bar = 100 µm. **E** Representative tissue sections (10 µm thick) of immunofluorescence staining showing DAPI (blue), elastin (autofluorescence, green), and SM-actin α (red). Scale bar = 100 µm. **F**–**H** Cumulative data showing the percentage of plaque area (**F**), the percentage of lumen area (**G**), and the ratio of cell number to media area (**H**). The data were analyzed with multiple Student’s *t*-tests (*n* = 9–10). *p < 0.05, ***p < 0.001.

Neointima formation involving SMC proliferation is considered to be the early stage of atherosclerosis [24]. Our finding that CFI-400945 inhibits neointima formation promoted us to explore its effect on the development of atherosclerosis. To focus on CFI-400945 effects on SMCs and avoid any other systemic side effects, we chose an atherogenic model induced by partial ligation of the carotid arteries in ApoE^−/−^ mice fed a high-fat diet and delivered CFI-400945 just around the carotid artery by hydrogel, as detailed in the Methods (Fig. 2A). Our ultrasound results one day after ligation indicated a successful partial but not a complete ligation (Fig. 2B, C). The LCA blood flow was significantly decreased compared with that in the RCA but not complete blocked. There was no significant difference in blood velocity between the vehicle control group and CFI-400945 group after surgery, showing the two groups had the same degree of initial partial ligation. After 18 days, the LCAs from mice treated with CFI-400945 were almost blocked, as shown by representative micrographs (Fig. 2D, upper panel). Oil Red O and HE staining showed that CFI-400945 significantly enhanced plaque formation in the LCA (Fig. 2D, middle and lower panel, and F) and significantly decreased lumen area (Fig. 2G). However, there was no significant difference in the aorta plaque area between the two groups (Fig. S1). In addition, our immunofluorescence staining revealed a significant decrease in SMCs within the vessel wall in response to CFI-400945 treatment (Fig. 2E, H). The two groups had no significant difference in mouse weight, total blood cholesterol levels, and triglyceride levels (Fig. S2A–C).

### CFI-400945 arrests vascular SMCs in the G2/M phase

**Fig. 3:**
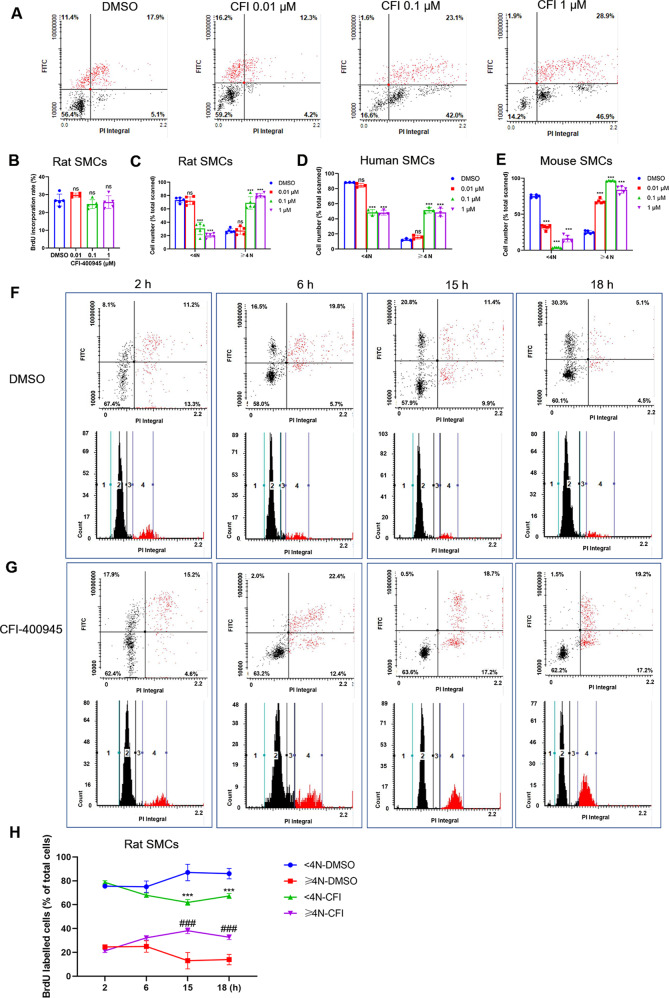
CFI-400945 arrests vascular SMCs in the G2/M phase. SMCs, cultured in Ham’s F12 medium containing 5% FBS, were treated with DMSO or different concentrations of CFI-400945 for 24 h, followed by BrdU pulse labeling (10 μM, 60 min) and BrdU staining with LSC analyses (**A**–**E**) or pulse-chase assay (**F**–**H**). **A** Representative LSC scattergrams showing the rates and location of BrdU-positive cells. **B** Cumulative data showing the rates of BrdU-positive cells (y-axis) in rat aortic SMCs; ns: not significant. **C** Cumulative data showing the distribution of cells with <4 N or ≥4 N DNA in rat aortic SMCs. **D**, Cumulative data showing the distribution of cells with <4 N or ≥4 N DNA in human aortic SMCs. **E** Cumulative data showing the distribution of cells with <4 N or ≥4 N DNA in mouse aortic SMCs. Data were analyzed by one-way ANOVA with Tukey’s post hoc; *n* = 5. ns: not significant. ***p < 0.001. For the BrdU pulse-chase assay, after BrdU pulse labeling, cells were treated with DMSO (**F**) or 10 µM CFI-400945 (**G**) for 2, 6, 15, and 18 h, as indicated. At each time point, cells were fixed for BrdU staining and LSC analyses. **F**, **G** Representative scattergrams (upper) and histograms (lower) showing the cell cycle positions of BrdU-labeled cells treated with DMSO (**F**) and CFI-400945 (10 μM, **G**). **H**, Cumulative data showing the distribution of cells with <4 N or ≥4 N DNA in the pulse-chase assay. Data were analyzed by two-way ANOVA; *n* = 3. ***p < 0.001, CFI-400945 versus DMSO (<4 N DNA). ^###^*p* < 0.001, CFI-400945 versus DMSO (≥4 N DNA).

We first determined cell cycle profiles of rat aortic SMCs with and without the presence of different concentrations of CFI-400945 using the BrdU incorporation and LSC assays (Fig. 3A). Interestingly, when the concentration of CFI-400945 was increased, it did not significantly affect BrdU incorporation rates (Fig. 3B) but significantly increased the number of cells with ≥4N DNA that were also BrdU-positive (Fig. 3C), suggesting the occurrence of SMC polyploidization. We also observed that CFI-400945 caused a considerable accumulation of cells with ≥4N DNA in human aortic SMCs (Fig. 3D) and mouse aortic SMCs (Fig. 3E).

To further investigate how CFI-400945 affects the cell cycle dynamics of SMCs, we conducted a BrdU pulse-chase assay, as described in the Methods. In the absence of CFI-400945, BrdU-labeled cells, as detected by LSC, moved from the S phase to the G2/M phase and then returned to G1/0 phases after mitosis. Specifically, 2 h after BrdU labeling, the percentage of BrdU-labeled 2N cells (left upper quadrant) treated with DMSO (control) decreased with a concomitant increase in BrdU-labeled 4 N cells (right upper quadrant) (Fig. 3F), suggesting that the BrdU-labeled cells entered G2/M phase. After 6 h, and particularly at 15 h, the percentage of BrdU-labeled 2N cells (left upper quadrant) markedly increased, indicating that the BrdU-labeled cells underwent mitosis or entered the G0/1 phase. In the presence of CFI-400945, however, most of the BrdU-labeled cells stayed in the G2/M phase or maintained 4N DNA content after 6 h. Most significantly, almost all the BrdU-labeled cells maintained ≥4N DNA content with tetraploidy or polyploidy after 15–18 h (Fig. 3G, H), suggesting that CFI-400945 treatment abolished all mitotic activity and induced polyploidization.

**Fig. 4:**
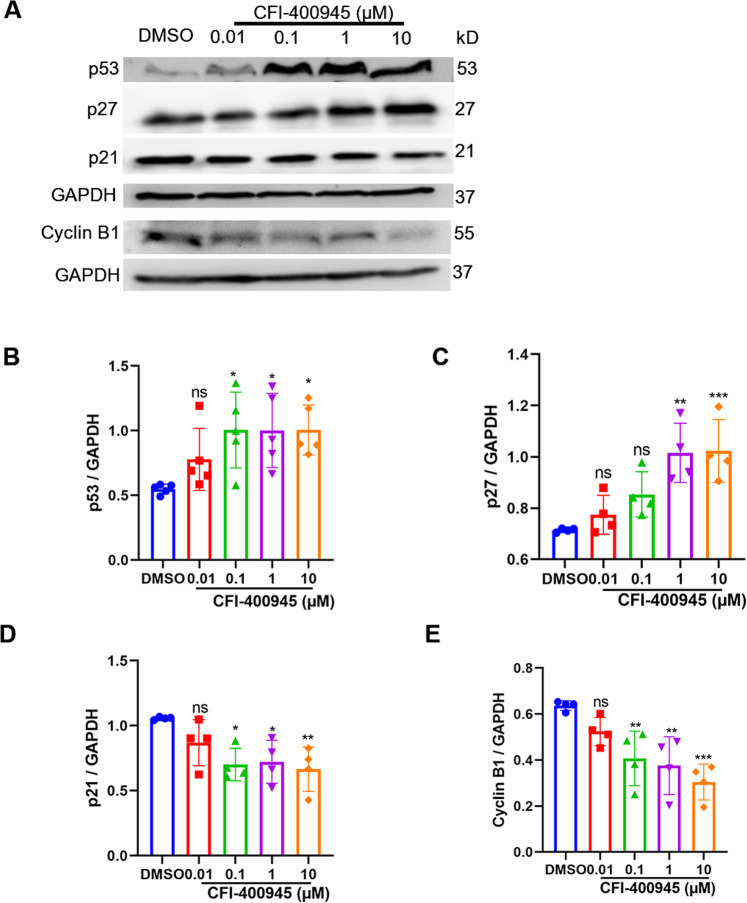
CFI-400945 upregulates p53 and p27 and downregulates p21 and cyclin B1 in vascular SMCs. Vascular SMCs, cultured in Ham’s F12 medium containing 5% FBS, were treated with DMSO or different concentrations of CFI-400945 (0.01, 0.1, 1, 10 μM) for 24 h, followed by extraction of total cell lysate proteins and Western blot (WB) assays. **A** Representative WB showing p53, p27, p21, cyclin B1 protein levels in human aortic SMCs treated with DMSO or different concentrations of CFI-400945 for 24 h. **B** Cumulative WB results showing the effect of CFI-400945 on the expression of p53 relative to GAPDH. **C** Cumulative WB results showing the effect of CFI-400945 on the expression of p27 relative to GAPDH. **D** Cumulative WB results showing the effect of CFI-400945 on the expression of p21 relative to GAPDH. **E** Cumulative WB results showing the effect of CFI-400945 on the expression of cyclin B1 relative to GAPDH. All data in B-E were expressed as mean ± SD and analyzed with one-way ANOVA with Tukey’s post hoc (*n* = 4–5). ns: not significant. *p < 0.05, **p < 0.01, and ***p < 0.001 versus DMSO control.

To further explore the biochemical mechanisms, we examined the expression levels of related proteins, such as p53, p27, p21, and cyclin B1. Supportively, our results showed that CFI-400945 concentration-dependently increased the expression levels of p53 and p27 (Fig. 4A–C), while decreased the p21 and cyclin B1 protein expression levels (Fig. 4A, D, E).

### CFI-400945 induces apoptosis and passaging-dependent senescence in SMCs

**Fig. 5:**
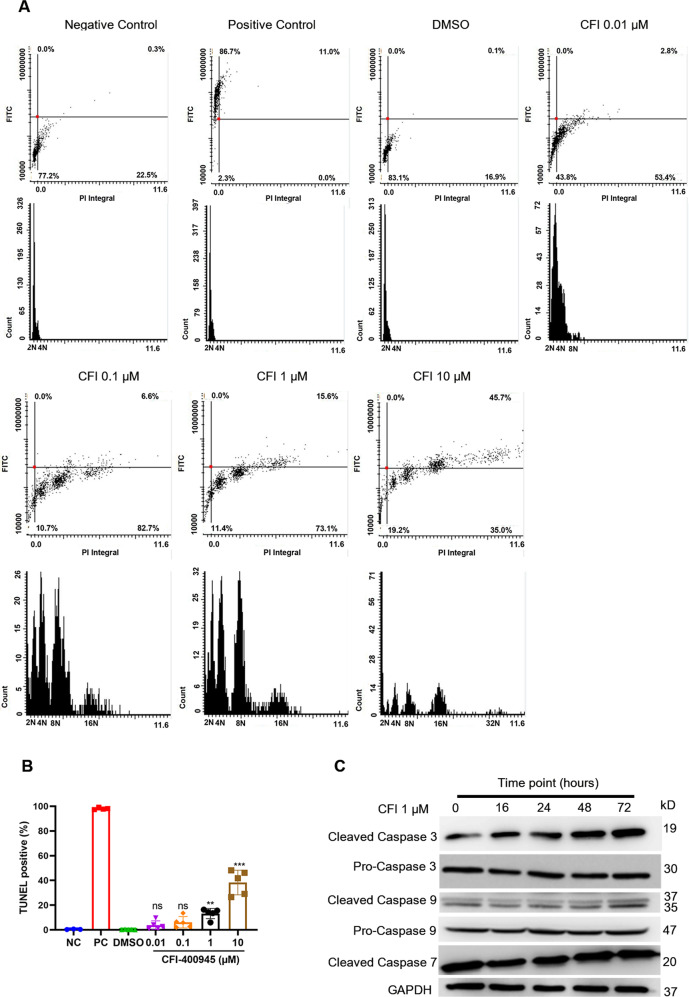
CFI-400945 induces apoptosis in vascular SMCs. SMCs were treated with DMSO or different concentrations of CFI-400945 for 72 h. **A**–**B** Cells were then fixed with 4% paraformaldehyde for the TUNEL assay. Negative control: cells were incubated with the labeling solution instead of the TUNEL reaction solution. Positive control: cells were treated with recombinant DNase I for 10 min to induce DNA strand breaks before labeling. **A** Representative LSC scattergrams (upper) and histograms (lower). **B** Cumulative data showing the percentages of TUNEL-positive cells (one-way ANOVA with Tukey’s post hoc; *n* = 5). **C** Representative WB. After treatment with and without CFI-400945 (1 μM) for different times, SMCs were harvested for WB analysis of caspase 3, 7, and 9 and their cleaved forms.

Since CFI-400945 treatment resulted in SMC polyploidization, we speculated that polyploidy of SMCs in response to CFI-400945 might lead to apoptosis or senescence as reported in cancer cells [10, 16]. To test this possibility, we performed a TUNEL assay for SMCs treated with or without CFI-400945. As expected, LSC scanning showed that at 72 h CFI-400945 treatment significantly increased polyploidy and TUNEL-positive cells (Fig. 5A, B). In addition, CFI-400945 treatment did not significantly affect the expression of smooth muscle contractile proteins, such as α-actin, SM22, and calponin (Fig. S3), suggesting it might not affect SMC differentiation. Furthermore, we observed that CFI-400945 treatment elevated the expression of cleaved caspase 3 and caspase 9, but not cleaved-caspase 7 (Fig. 5C), suggesting the involvement of caspase activation in CFI-400945-induced apoptosis in SMCs. Nevertheless, our SA-β-gal staining did not show significant SMC senescence in response to CFI-400945 treatment for 72 h (Fig. S4A, B). However, SA-β-gal positive cells were significantly increased after passaging during the treatment period (Fig. S4C, D). To further confirm our results, we also performed the TUNEL assay and Ki67 immunofluorescence in vivo. As the results showed (Fig. S5), after treatment with CFI-400945 for 21 days in the neointima model, a few TUNEL-positive cells were detected (Fig. S5A), and no Ki67-positive cells were detected in the CFI-400945 group (Fig. S5B), which was consistent with the in vitro study.

**Fig. 6:**
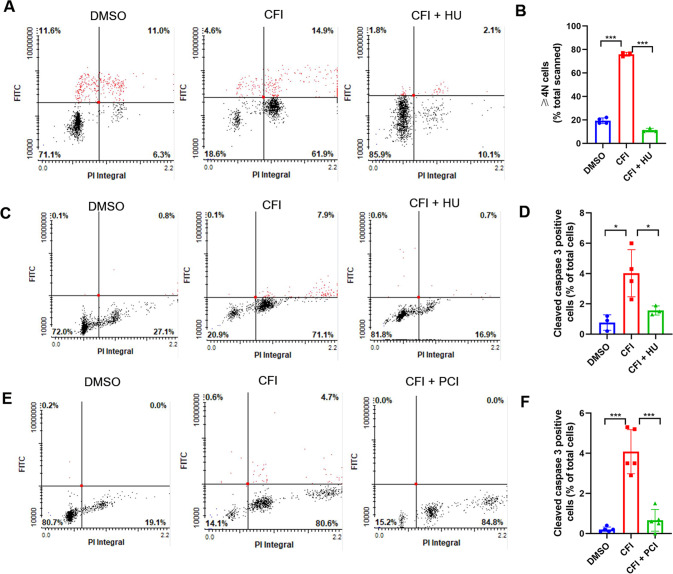
CFI-400945 causes SMC polyploidization, leading to apoptosis. Vascular SMCs were pre-treated with hydroxyurea (HU, 100 μg/ml) or pan-caspase inhibitor (PCI) Z-VAD-FMK (25 μM) for 1 h, followed by CFI-400945 treatment for 24 h. **A** Representative LSC scattergrams of cells fixed for BrdU staining. **B** Cumulative data showing the distribution of cells with ≥4 N DNA. **C**, Representative LSC scattergrams of cells fixed for cleaved caspase 3 staining. **D** Cumulative data showing the percentage of cleaved caspase 3-positive cells. **E** Representative LSC scattergrams of cells fixed for cleaved caspase 3 staining. **F** Cumulative data showing the percentage of cleaved caspase 3-positive cells. Data were analyzed by one-way ANOVA with Tukey’s post hoc; *n* = 3–5. ***p < 0.001.

To further investigate the cause-effect relationship between SMC polyploidy and apoptosis, we used hydroxyurea to inhibit DNA synthesis and polyploidization and then determine apoptosis. As anticipated, our results showed that hydroxyurea significantly decreased the accumulation of cells with ≥4N DNA caused by CFI-400945 treatment (Fig. 6A, B), which was accompanied by a decrease in cleaved caspase 3-positive cells (Fig. 6C, D). Next, we used a pan-caspase inhibitor (PCI) Z-VAD-FMK to block caspase activation, followed by determining polyploidy. The result showed that PCI significantly decreased cleaved caspase 3 positive cells but did not influence the number of polyploid cells caused by CFI-400945 (Fig. 6E, F). These findings suggest that CFI-400945 causes SMC polyploidy, subsequently leading to apoptosis.

### CFI-400945 causes dysregulation of centrosome duplication

**Fig. 7:**
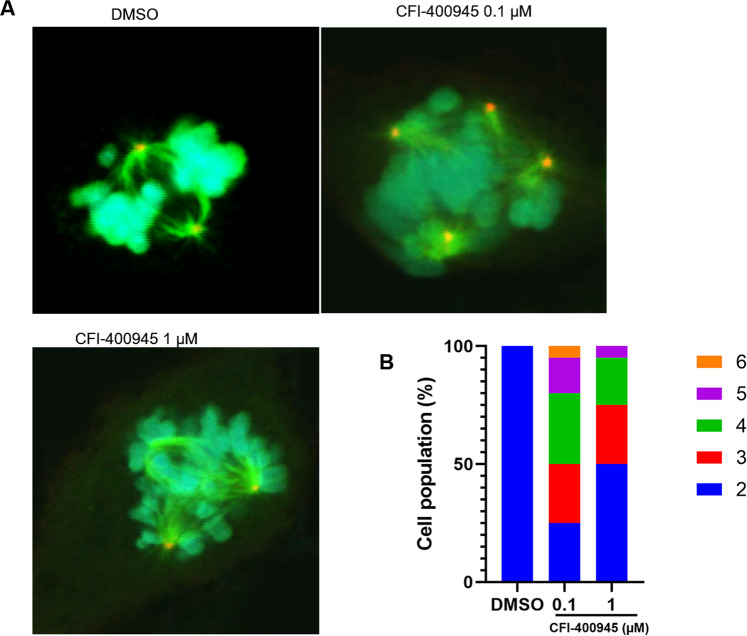
CFI-400945 causes dysregulation of centrosome duplication. SMCs were treated with DMSO or different concentrations of CFI-400945 for 24 h, followed by overnight fixation with 100% methanol. Cells were stained for α-tubulin and γ-tubulin and the nuclei counterstained with DAPI. **A** Representative micrographs acquired with confocal microscopy showing the mitotic spindle (α-tubulin, green), centrosomes (γ-tubulin, red), and chromosomes (DNA, blue) of mitotic cells. **B** Percentages of cell populations are displayed according to centrosome number before or after CFI-400945 treatment.

CFI-400945 is known to target PLK4 and regulate centriole duplication of cancer cells [25]. Thus, we evaluated the centrosome number and mitotic status of SMCs treated with CFI-400945 or DMSO by staining for α-tubulin and γ-tubulin and counterstained the nuclei with DAPI. As shown by representative cell images (Fig. 7A), CFI-400945 caused supernumerary centrosome number and multipolar spindles. Cell populations with supernumerary centrosomes were increased in response to CFI-400945 treatment (Fig. 7B), which may cause mitotic failure and polyploidization.

## Discussion

In this study, we demonstrated that CFI-400945, a selective inhibitor of PLK4, inhibited neointima formation of mouse carotid arteries resulting from complete carotid ligation but accelerated partial carotid ligation-induced atherosclerosis in ApoE^−/−^ mice fed a high-fat diet. Mechanistically, we found that treatment with CFI-400945 caused dysregulated centrosome duplication of vascular SMCs, arrested cells in the G2/M phase, and induced polyploidy and subsequent apoptosis. It is well known that PLK4, a serine/threonine protein kinase, is aberrantly expressed in cancer cells but normally has a low abundance and is only present in proliferating normal tissues [26]. Notably, vascular SMCs, unlike skeletal and cardiac muscle that are terminally differentiated, have phenotype plasticity [27]. They proliferate after losing a contractile phenotype and acquiring a synthetic phenotype [27]. Thus, it is reasonable that PLK4 inhibitor CFI-400945 has significant effects on SMC proliferation and proliferative vascular disease. The current study is the first to demonstrate a regulatory role for PLK4 in vascular SMCs. Also, our studies have revealed several novel findings in terms of CFI-400945 and the pathogenesis of proliferative vascular diseases.

CFI-400945 was discovered as a cancer drug by targeting PLK4, which has a pivotal role in regulating centriole duplication [28–31]. Besides regulating centriole duplication, phosphorylated PLK4 was also reported to localize to the centrosome, kinetochore, cleavage furrow, and midbody during different phases of the cell cycle with catalytic activity, suggesting its fundamental role in cell abscission during cytokinesis [11]. Furthermore, it was found that CFI-400945 causes supernumerary centrosomes and multipolar mitotic defects [10, 26]. In vascular SMCs, we also observed that CFI-400945 induced dysregulation of centrosome duplication, likely through similar mechanisms identified in cancer cells. For example, under confocal microscopy, we found that CFI-400945 caused extra centrosomes and multipolar mitosis, eventually leading to mitotic defects. However, it appears that there are some differences in response to this compound between vascular SMCs and cancer cells. In cancer cells, CFI-400945 treatment upregulated the expression of p53, an essential regulator of the G2/M checkpoint [32, 33], which activates cell cycle inhibitors, including p21 and p27 [34], and arrests the cell cycle. Our study revealed that CFI-400945 increased p27 protein levels but decreased p21 expression, suggesting that CFI-400945 treatment caused SMC cell cycle arrest mainly through the p53/p27 pathway.

Unscheduled cyclin B1 degradation is involved in the predisposition of SMCs to polyploidy, as described by us and others [35, 36]. Consistently, our data also showed that cyclin B1 levels decreased after CFI-400945 treatment, which is also supportive of G2/M phase arrest. It is known that gradual cyclin B1 degradation in prolonged spindle checkpoint activation causes cells to exit mitosis. Cells, after mitotic slippage, enter G1 as tetraploid cells and may continue through the cell cycle, and undergo apoptosis or senescence [37]. This speculation is supported by our BrdU pulse-chase assay showing that CFI-400945 induced G2/M phase arrest and polyploidization. However, for an unknown reason, CFI-400945-treated cells underwent significant senescence after subculture. Thus, subculture may accelerate the senescence of polyploid SMCs. Previous evidence suggests that SMC polyploidization precedes apoptosis and senescence [23, 38]; however, whether CFI-400945-induced polyploidization directly results in apoptosis has not been previously reported.

In various cancer cells, treatment with CFI-400945 induces polyploidization [10, 12, 13]. It is interesting to note that polyploidy and aneuploidy are significant features of tumorigenesis [14, 15]. It was not understood how CFI-400945 induces polyploidy but inhibits tumorigenesis. Our studies using LSC, the instrument of choice for polyploid studies [39], revealed that CFI-400945-induced polyploidization results in SMC apoptosis, as indicated by TUNEL assay and an increase in cleaved caspase 3 and caspase 9. This cause-effect relationship was supported by: 1) apoptosis occurred after the appearance of polyploidy; 2) inhibition of DNA synthesis, which is known to inhibit polyploidization, prevented apoptosis; 3) inhibition of apoptosis by pan-caspase inhibitor (Z-VAD-FMK) did not affect polyploidization. Importantly, the mechanism we proposed here for SMCs may be also applied to CFI-400945 treatment of cancer and explains why CFI-400945 induces polyploidy to inhibit tumorigenesis. For vascular SMCs, however, such an effect of CFI-400945 may have dual implications to confer the differential effects of CFI-400945 on neointima formation and atherosclerosis.

First, it must be noted that SMC polyploidization in aortas is well documented in aging and hypertensive animals and humans [18–20], but the interrelationship between aging or hypertension and SMC polyploidization remains poorly understood. However, it is well known that aging and hypertension are the primary etiologies of atherosclerosis. Our studies have shown that CFI-400945 treatment results in SMC polyploidization and accelerated atherosclerosis, strongly suggesting a role of SMC polyploidy in the pathogenesis of atherosclerosis. The key evidence to support this speculation is our observation that polyploidization of SMCs leads to apoptosis. It is conceivable that apoptosis of SMCs resulting from polyploidization causes the release of proinflammatory cytokines and many other factors [40], including IL-1α and IL-1β [41], consequently accelerating atherosclerosis in the presence of atherogenic factors. Given that SMC apoptosis contributes to a thin fibrous cap, enlarged necrotic core, and macrophage infiltration into the cap, promoting multiple features of vulnerable plaques [42], it is reasonable that CFI-400945-induced SMC apoptosis accelerates atherogenesis.

Furthermore, in our atherosclerosis model, the atherogenic factors include flow turbulence due to partial ligation and hyperlipidemia in ApoE^−/−^ mice. The pathogenesis of atherosclerosis, when compared with neointima formation, is a much more complicated process, involving not only SMC dedifferentiation and proliferation but also the recruitment of macrophages and other inflammatory cells into the intimal layer for the inflammatory response. It is a limitation that we cannot rule out the possibility of CFI-400945 effects on other cells. However, we think PLK4 inhibitor increased atherosclerosis at least partly by SMC polyploidization and apoptosis for the following two reasons. First, we used an accelerated atherosclerosis model induced by partial ligation, which is characterized by accelerated endothelial dysfunction following SMC migration and proliferation. PLK4 is aberrantly expressed in cancer cells but normally has a low abundance and is only present in proliferating normal tissue. Also, vascular SMCs are not terminally differentiated and proliferate after losing a contractile phenotype [27]. Together with the neointima model in vivo study and the SMC in vitro study, it is conceivable that, in the absence of those factors existing in atherosclerosis, treatment with CFI-400945 inhibits neointima formation through its effects on SMCs. Second, to avoid other side effects as much as possible, we choose to deliver CFI-400945 to the ligation by hydrogel instead of systemic application. Thus, vascular wall cells including SMCs are directly influenced by CFI-400945 instead of inflammatory cells like macrophages. Taken together, we attributed the increased atherosclerosis at least partly to SMC polyploidization and apoptosis induced by CFI-400945. Of course, we cannot rule out the possible effect of CFI-400945 on macrophages and other cells according to current results and a more complete mechanism needs to be further investigated.

In sum, our study has demonstrated that CFI-400945, an anticancer drug that inhibits PLK4, significantly blocks intimal hyperplasia of mouse carotid arteries but increases atherosclerosis in vivo and the underlying mechanism may involve the induction of SMC polyploidization and subsequent apoptosis. Our findings have shed light on the potential uses of cancer drugs such as CFI-400945 for proliferative vascular disease and the interrelationship between neointima lesion and progression of atherosclerosis.

## Materials and Methods

### Materials

Male C57BL/6J mice were purchased from the Jackson Laboratory (Bar Harbor, ME, USA). Human aortic SMCs (CRL-199) were purchased from the American Type Culture Collection (ATCC; Manassas, VA, USA) and authenticated by STR profiling. Primary rat SMCs were cultured from the aorta of Sprague Dawley (SD) rats (Charles River, Canada) and primary mouse SMCs were cultured from the aorta of C57BL/6J mice (Jackson Laboratory, Canada). CFI-400945 (#7552) and Z-VAD-FMK (#7023) were purchased from SelleckChem (Burlington, ON, Canada). Collagenase Type II (#9001-12-1), Dulbecco’s modified eagle medium (DMEM)-F12 medium (#2323609), Ham’s F12 nutrient medium (#11330), 10000 units/mL penicillin (#15140122), 10000 units/mL streptomycin solution (#15140122), and fetal bovine serum (FBS; #A4766801) were bought from Thermo Fisher Scientific (Ottawa, ON, Canada). 5-bromodeoxyuridine (BrdU; #B5002), propidium iodide (PI; #P4170), hydroxyurea (HU; #H8627), and hydrogel (#P2443) were purchased from Sigma-Aldrich Canada (Oakville, ON, Canada). BrdU mouse monoclonal antibody (#MA1-19213) was bought from Thermo Fisher Scientific, and the senescence-associated β-galactosidase (SA-β-gal) staining kit (No. 9860S) from Cell Signaling Technology (Whitby, ON, Canada). The in-situ Cell Death Detection Kit, terminal transferase dUTP nick end labeling (TUNEL) kit was purchased from Roche (Mississauga, ON, Canada). Primary antibodies for p53 (#2524), p27 (#3686), cyclin B1 (#12231), caspase 3 (#14220), cleaved-caspase 3 (#9664), caspase 7 (#12827), cleaved-caspase 7 (#8438), caspase 9 (#9508), cleaved-caspase 9 (#7237), GAPDH (#5174), and goat anti-rabbit secondary antibody (#7074P2) were purchased from Cell Signaling Technology (Whitby, ON, Canada). Ki67 antibody (#NB600-1252) was bought from NOVUS (Toronto, ON, Canada). Antibodies for p21 (#10355-1-AP) were purchased from Proteintech (Chicago, IL, USA) and goat anti-mouse secondary antibodies (#A9044) from Sigma-Aldrich Canada. An enhanced chemiluminescence (ECL) solution was bought from Bio-Rad (Mississauga, ON, Canada). Total cholesterol kit (#999-02601) and Triglyceride kit (#290-63701) were purchased from FUJIFILM (Lexington, MA, USA).

### Neointima formation in mouse carotid arteries

All animals were maintained at room temperature (approximately 22 °C) with food and water available *ad libitum*. C57BL/6J male mice at 12 weeks of age were randomly divided into vehicle control and CFI-400945 treatment group (the investigator was not blinded to the group allocation) and received the left carotid artery (LCA) complete ligation, as we previously described [23]. Briefly, the animals were anesthetized with 5% isoflurane. Anesthesia was confirmed by no reflex response to hind feet pinching and was sustained with 1% isoflurane at 20 ml/min during the surgery. Then, the LCA was dissected and ligated proximally to the bifurcation of the internal and external carotid arteries. CFI-400945 (10 µM) was applied through perivascular application of 20% hydrogel at the ligation site. Control mice received 20% hydrogel with the same volume of DMSO. Eighteen days after ligation, the animals were euthanized using a CO_2_ chamber and perfused, followed by isolation of carotid arteries. Arterial tissues were fixed with 4% paraformaldehyde at 4 °C overnight and then embedded in paraffin. Serial 5-μm-thick paraffin sections were prepared, covering the region within 2 mm proximal to the ligation site, followed by hematoxylin and eosin (HE) staining. All animal studies were approved by the Institutional Animal Care and Use Committees at the University of Calgary and were performed in accordance with the US National Institutes of Health guidelines.

### Atherosclerosis resulting from partial carotid ligation in ApoE^−/−^ mice fed a high-fat diet

5-month-old ApoE^−/−^ male mice were randomly divided into vehicle control and CFI-400945 treatment group (the investigator was not blinded to the group allocation). Partial carotid ligation was performed in as previously described [43]. Anesthesia was carried out as detailed in complete ligation. Briefly, the three branches in the LCA, including the left external carotid artery (ECA), left internal carotid artery (ICA), and occipital artery (OA), were ligated by 9–0 Ethalon suture while leaving the superior thyroid artery (STA) intact. All ApoE^−/−^ mice with partial carotid ligation received CFI-400945 (10 µM) through perivascular application of 20% hydrogel at the ligation site or 20% hydrogel with DMSO vehicle alone as a control. The LCA competency of all mice was confirmed with ultrasound the day after ligation and re-examined 2 weeks after ligation. All the mice were fed a high-fat atherogenic diet (21% milk fat, 1.25% cholesterol). After 18 days, the mice were euthanized using a CO_2_ chamber, followed by collecting blood samples, perfusion of the arteries to remove the residual blood, fixation with paraformaldehyde, and embedding with OCT (optimal cutting temperature, -20 °C). Arterial tissue sections (10-μm-thick) were prepared for Oil Red O, HE, or immunofluorescence staining.

### Cell culture and isolation of primary rat and mouse aorta SMCs

Human SMCs were cultured and maintained in DMEM-F12 medium containing 100 U/mL penicillin, 100 U/mL streptomycin, and 10% FBS. Treatment of cells with CFI-400945 and vehicle or other reagents were indicated in each respective experiment. Primary rat SMCs were cultured from rat aortas, according to a previously described method [44]. Briefly, the aortas were isolated with fat tissues and the adventitia removed and the intima scraped. The aortas were cut into approximately 1 mm^3^ sections, followed by explantation for about 2 h before the addition of DMEM-F12 medium with 20% FBS. After reaching subconfluence, cells were subcultured with DMEM-F12 medium containing 10% FBS and penicillin/streptomycin.

Primary mouse SMCs were cultured from mouse aortas, as previously described [45]. Briefly, the aortas were cut into small pieces that were approximately square and 1–2 mm with adventitia removed, digested with 1.5 mg/ml type II collagenase, and placed in a standard tissue culture incubator at 37 °C, 5% CO_2_ for four to six hours. Complete culture medium was added to stop digestion and samples were centrifuged at 300 × g for 5 min. Cells were then resuspended and cultured with DMEM/F12 containing 10% FBS.

Immunofluorescence staining was performed on isolated SMCs and the rate of α-SMA positive cells above 80% suggested corrected SMCs were extracted. And cells had no mycoplasma contamination.

### 5-bromodeoxyuridine (BrdU) incorporation and pulse-chase assays

SMCs were seeded on coverslips, followed by treatment with different concentrations of CFI-400945 or one concentration for different periods. At the end of treatment, cells were labeled with BrdU (10 μM) for 60 min, followed by fixation with 80% ethanol and immunostaining with BrdU mouse monoclonal antibody (1:200) and Alexa Fluor 488-conjugated anti-mouse secondary antibody (1:500). The nuclei were counterstained with PI (5 μg/ml), followed by mounting with 90% glycerol containing PI (5 μg/ml). BrdU incorporation was analyzed with a Laser Scanning Cytometer (LSC; CompuCyte Corp, Cambridge, MA, USA), a microscope-based cytometer, as we previously described [46].

Cell cycle dynamics were analyzed using a BrdU pulse-chase assay as we previously described [47]. In brief, all cells on the coverslips were pulse-labeled with BrdU (10 μM) for 60 min. Cells were fixed right after labeling and set as the 0 h time point. Other cells were returned to the incubator for culture in the absence or presence of CFI-400945 (10 μM). At different time points (2, 6, 15, 18 h), a fraction of cells was retrieved for fixation. Cells were then processed for LSC analyses of BrdU incorporation and cell cycle profile, as described above for the BrdU incorporation assay.

### Senescence-associated β-galactosidase (SA-β-gal) staining

Vascular SMCs were treated with different concentrations of CFI-400945 (0, 0.01, 0.1, 1, 10 µM) for 72 h, followed by β-gal staining using an SA-β-gal staining kit. In brief, cells were fixed with 4% paraformaldehyde for 15 min after being rinsed with PBS and then incubated with freshly prepared SA-β-gal staining solution (pH 6.0) at 37 °C for 18 h. The proportion of SA-β-gal-positive cells in blue was determined based on the total number of counted cells.

### Terminal transferase dUTP nick end labeling (TUNEL) assay

SMCs were cultured on coverslips, followed by treatment with different concentrations of CFI-400945 (0, 0.01, 0.1, 1, 10 µM) for 72 h. After treatment, apoptosis was detected with an in-situ Cell Death Detection Kit. Briefly, cells were rinsed with PBS and fixed with 4% paraformaldehyde at room temperature for 60 min. Cells were then incubated with 50 µL of freshly prepared TUNEL reaction mixture at 37 °C in the dark for 60 min. TUNEL-positive cells were then detected and analyzed with LSC scanning.

### Immunochemistry staining

For observing mitotic spindle and centrosomes, an immunofluorescence assay was used to detect α-tubulin and γ-tubulin in SMCs. Briefly, cells grown on coverslips were fixed in methanol overnight at −20 °C, followed by incubation overnight with antibodies against α-tubulin (1:250) or γ-tubulin (1:300). Then, cells were incubated with goat anti-rabbit Alexa Fluor 488 (1:400) or anti-mouse rhodamine red secondary antibodies (1:400), respectively, for 60 min at 37 °C. The nuclei were counterstained with DAPI. Mitotic spindles and centrosomes were analyzed using a confocal microscope (FV10i Microscope, Olympus, Tokyo, Japan).

### Western blot analysis

Cultured SMCs with various treatments were harvested and lysed using radioimmunoprecipitation assay (RIPA) buffer, followed by protein extraction and measurement of protein concentration using the BCA Protein Assay Kit. The same amount of protein from different cell lysates were separated using 10% or 12% sodium dodecyl sulfate (SDS)-polyacrylamide gel electrophoresis (PAGE), followed by transfer to nitrocellulose membranes. The membranes were blocked with 5% fat-free milk and then incubated overnight at 4 °C with various primary antibodies targeting p53 (1:1000), p27 (1:1000), p21 (1:1000), cyclin B1 (1:1000), caspase 3 (1:1000), cleaved-caspase 3 (1:1000), caspase 7 (1:1000), cleaved-caspase 7 (1:1000), caspase 9 (1:1000), cleaved-caspase 9 (1:1000), and GAPDH (1:1000). After incubation with goat anti-rabbit secondary antibody (1:5000) or goat anti-mouse secondary antibody (1:50000), immunoreactive bands were analyzed using ECL and an Image Quant LAS-4000 camera (GE Healthcare, Mississauga, ON, Canada). The relative abundance of proteins was analyzed with ImageJ software.

### Statistical analysis

All data collected from at least three independent experiments were recorded as mean ± standard deviation (SD). Sample size was not predetermined by statistical method. GraphPad Prism v8.0 (GraphPad, San Diego, CA, USA) was used for data analysis. Student’s *t*-test or one-way analysis of variance (ANOVA) with Tukey’s post-hoc test was used for normally distributed data in group pairs or multiple groups. The Mann-Whitney *U* test or the Kruskal-Wallis test was used for non-normally distributed data in group pairs or multiple groups. A difference with *p* < 0.05 was considered statistically significant.

## Supplementary information

Supplemental figures

Original Data File

## Data Availability

Preliminary data can be acquired by requesting from the corresponding author.
