# Supplementary material for: Polo-like kinase 4 inhibitor CFI-400945 inhibits carotid arterial neointima formation but increases atherosclerosis
Source: Cell Death Discov. 2023 Feb 7;9:49. doi: 10.1038/s41420-023-01305-4 (PMC9905587; doi:10.1038/s41420-023-01305-4)

**Supplemental Figures**

**Figure S1 Perivascular application of CFI-400945 does not increase plaque in aorta root in ApoE ^-/^**^-^ **mice with carotid artery partial ligation.** ApoE ^-/-^ mice received partial ligation and were euthanized after 18 days. **A**, Oil Red O staining was used to observe atherosclerosis plaque in the aorta root. **B**, Cumulative data showing the percentage of plaque area. All the data were expressed as mean ± SD and analyzed with the Student’s *t*-test, n=5. ns: not significant.

**Figure S2 Perivascular application of CFI-400945 does not affect mouse body weight, blood** **cholesterol and triglyceride levels.** 18 days after partial ligation, ApoE ^-/-^ mice were sacrificed, and blood samples were collected for analyses of total cholesterol and triglyceride using Cholesterol E and Triglyceride kits, respectively. **A**, Mouse body weight at the end of experiments. **B** and **C**, Total cholesterol and triglyceride levels, respectively. All the data were expressed as mean ± SD and analyzed with the Student’s *t*-test, n=9-10. ns: not significant.

**Figure S3 CFI-400945 does not modulate vascular SMC differentiation.**

Cells were treated with DMSO or different concentrations of CFI-400945 for 24 h. Western blot was performed to assess α-actin, SM 22, and calponin protein expression in SMCs treated with DMSO or different concentrations of CFI-400945. GAPDH was used as loading control.

**Figure S4 CFI-400945 treatment induces senescence of vascular SMCs after subculture.** Senescence of SMCs treated with or without (DMSO control) CFI-400945 was detected using SA-β-gal staining. **A**, Representative micrographs of SA-β-gal staining of SMCs with and without different concentrations of CFI-400945 (0.01, 0.05, 0.1, 1, and 10 μM) for 72 h. **B**, Cumulative data showing the percentages of SA-β-gal positive cells. **C**, Cultured SMCs were first treated with CFI-400945 (1 μM) for 48 h, followed by subculture for 24 h. SMCs were then fixed for SA-β-gal staining. Red arrows indicate SA-β-gal positive cells. **D**, Statistical data showing the percentages of SA-β-gal positive cells. All the data were expressed as mean ± SD and analyzed with the Student’s *t*-test. ^***^p<0.001 versus DMSO control.

**Figure S5 Perivascular application of CFI-400945 induced apoptosis *in vivo.***

C57BL/6 mice received complete ligation and were euthanized after 18 days. **A**, TUNEL staining was used to determine apoptotic cells. DAPI, Blue. TUNEL, Green. **B**, Ki-67 immunofluorensce was used to detect SMC proliferation. DAPI, Blue. Ki67, Green. Positive control, Glioma tissue.

**Figure S1**

**
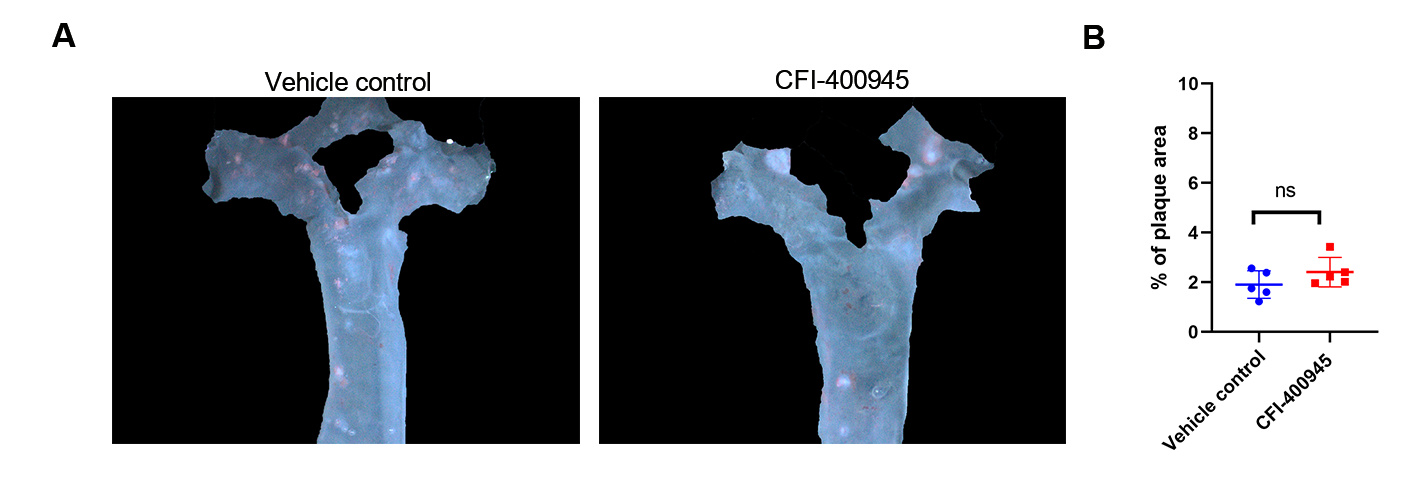
**

**Figure S2**

**
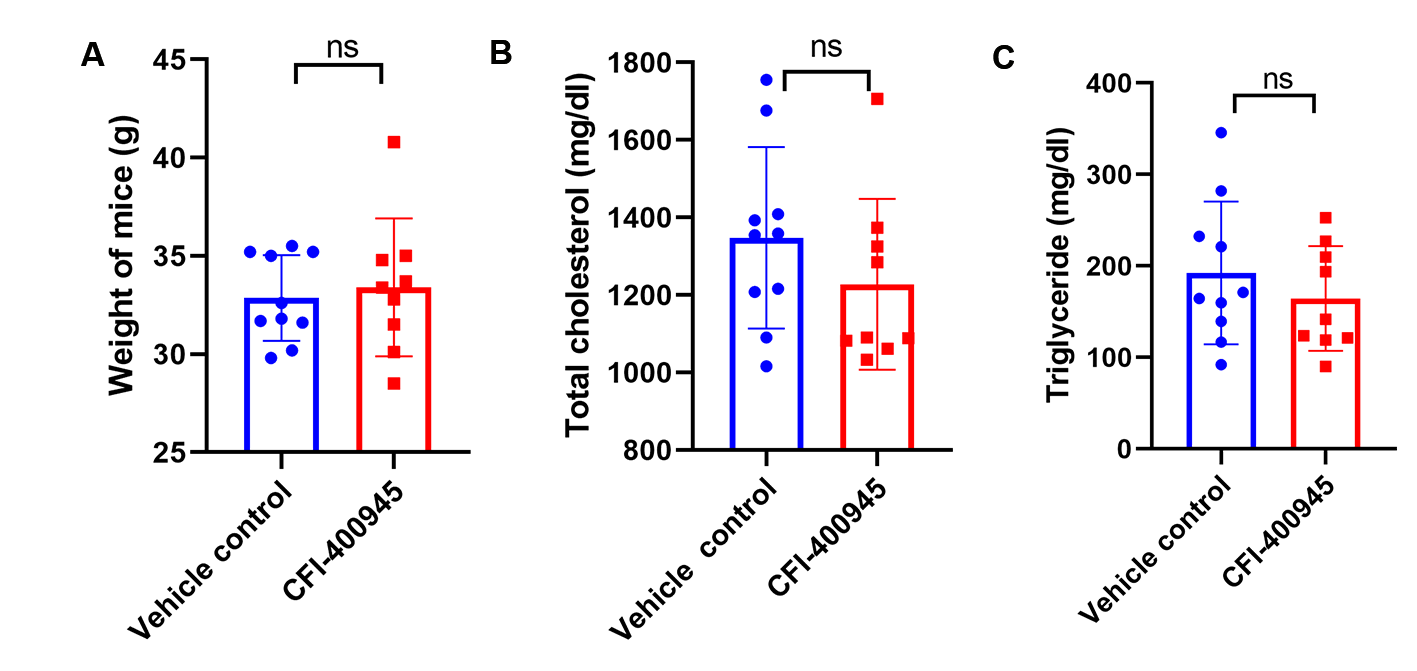
**

**Figure S3**


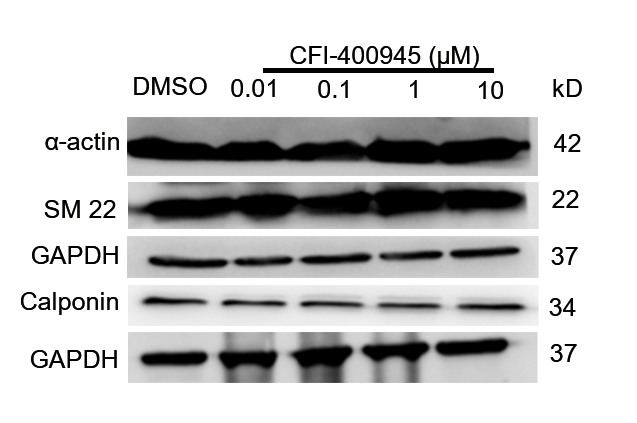


**Figure S4**


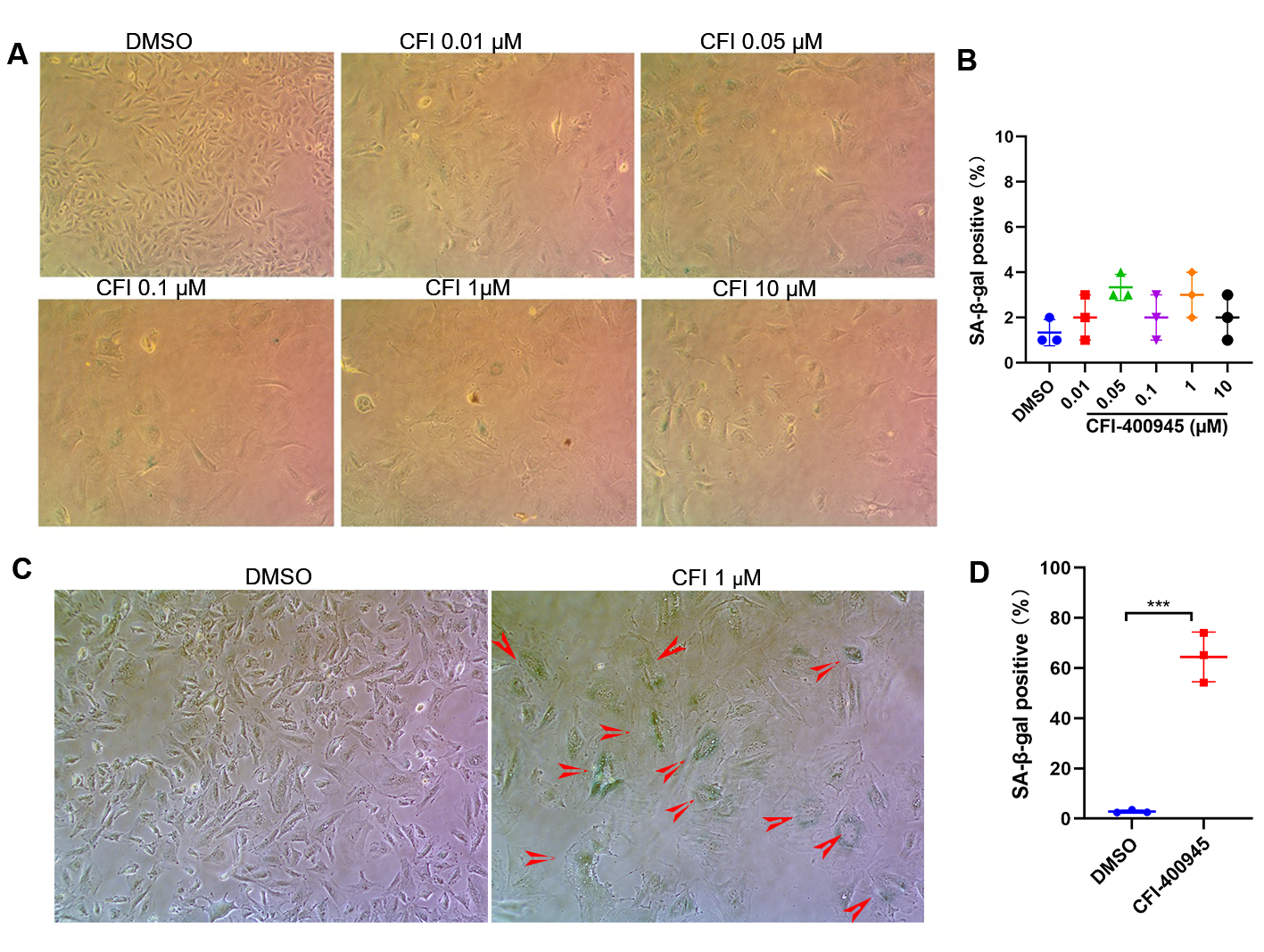


**Figure S5**


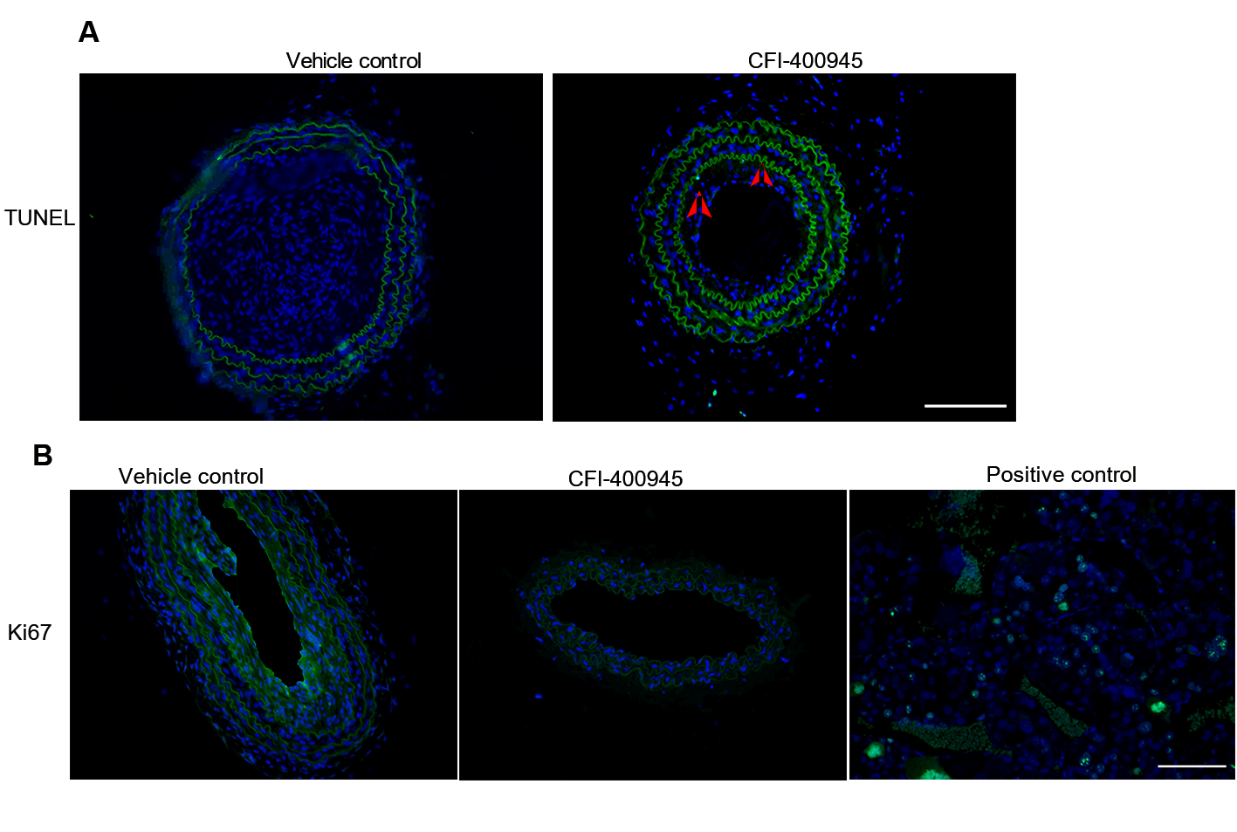

Supplement: Supplementary file 1 — Supplemental figures [file 41420_2023_1305_MOESM1_ESM.docx]
