# Supplementary material for: Polo-like kinase 4 inhibitor CFI-400945 inhibits carotid arterial neointima formation but increases atherosclerosis
Source: Cell Death Discov. 2023 Feb 7;9:49. doi: 10.1038/s41420-023-01305-4 (PMC9905587; doi:10.1038/s41420-023-01305-4)

Figure 4A

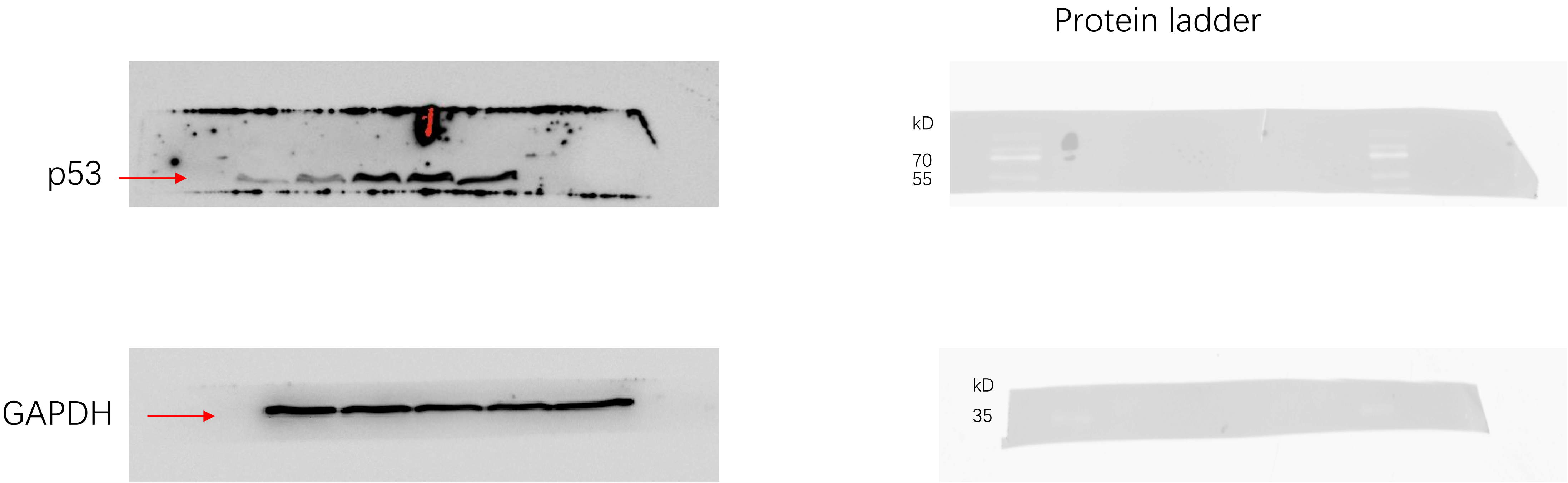

Figure 4A

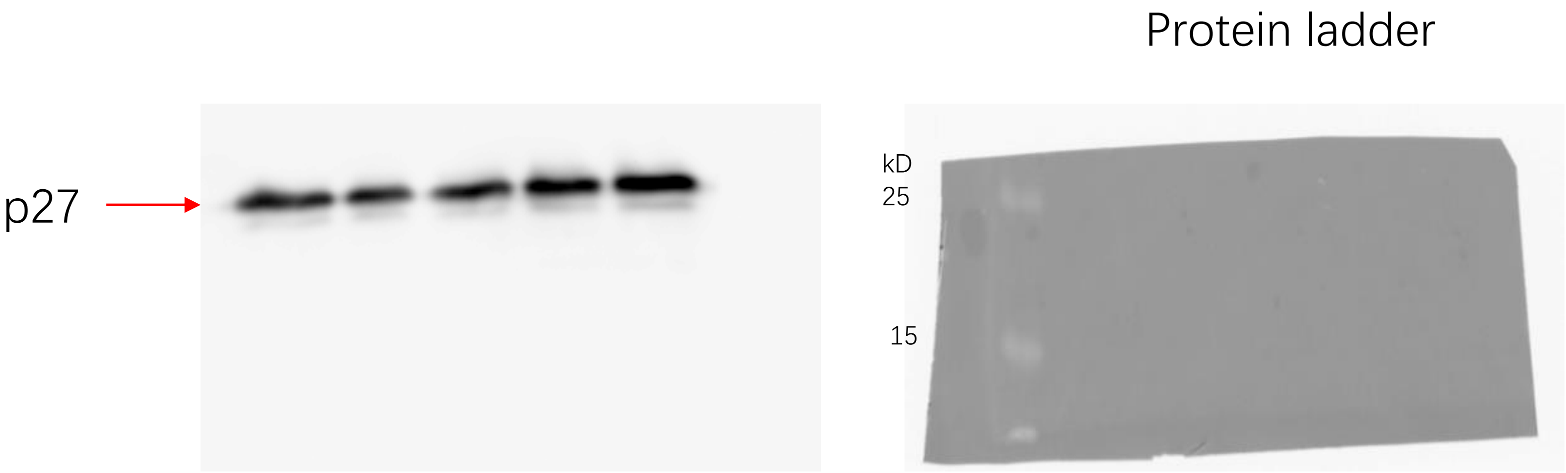

Figure 4A

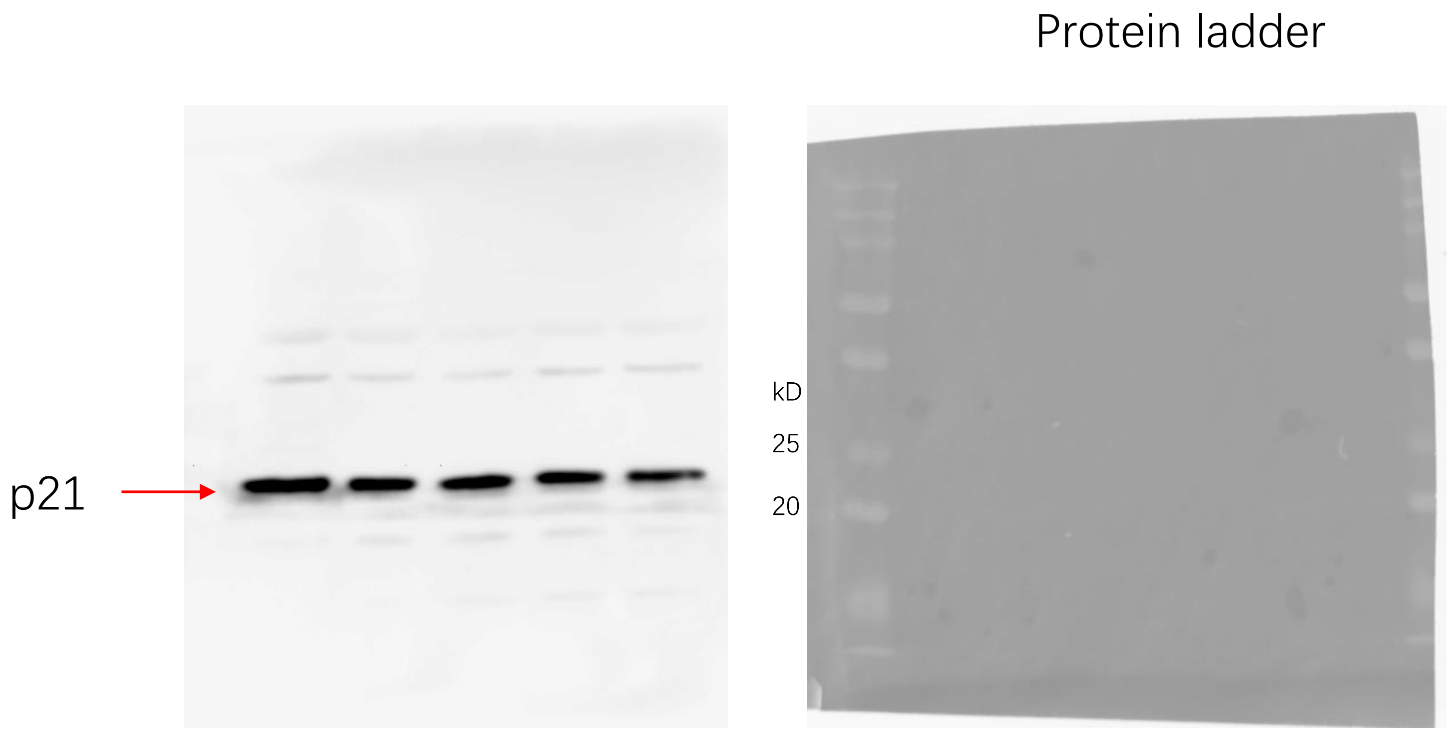

Figure 4A

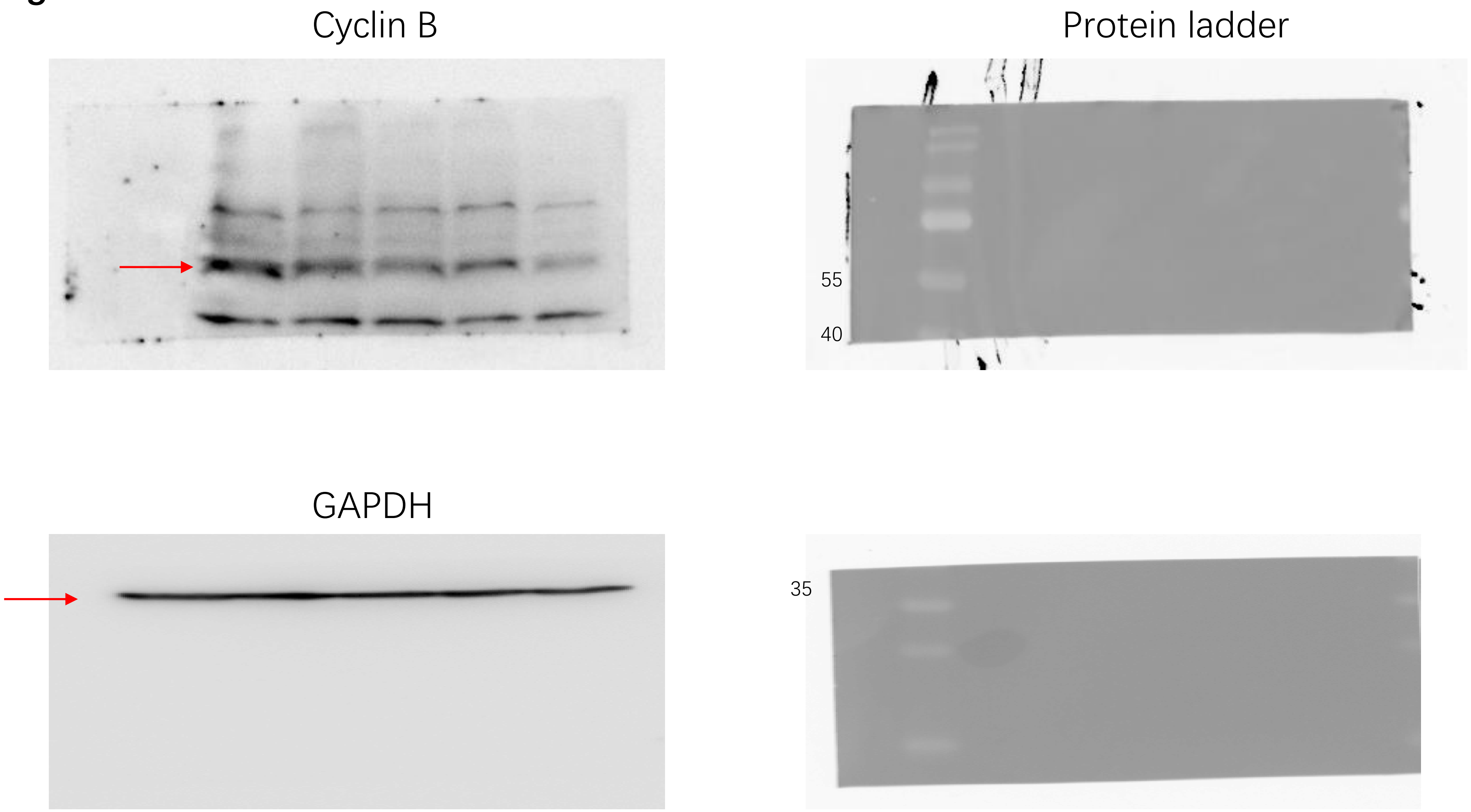

Figure 5C

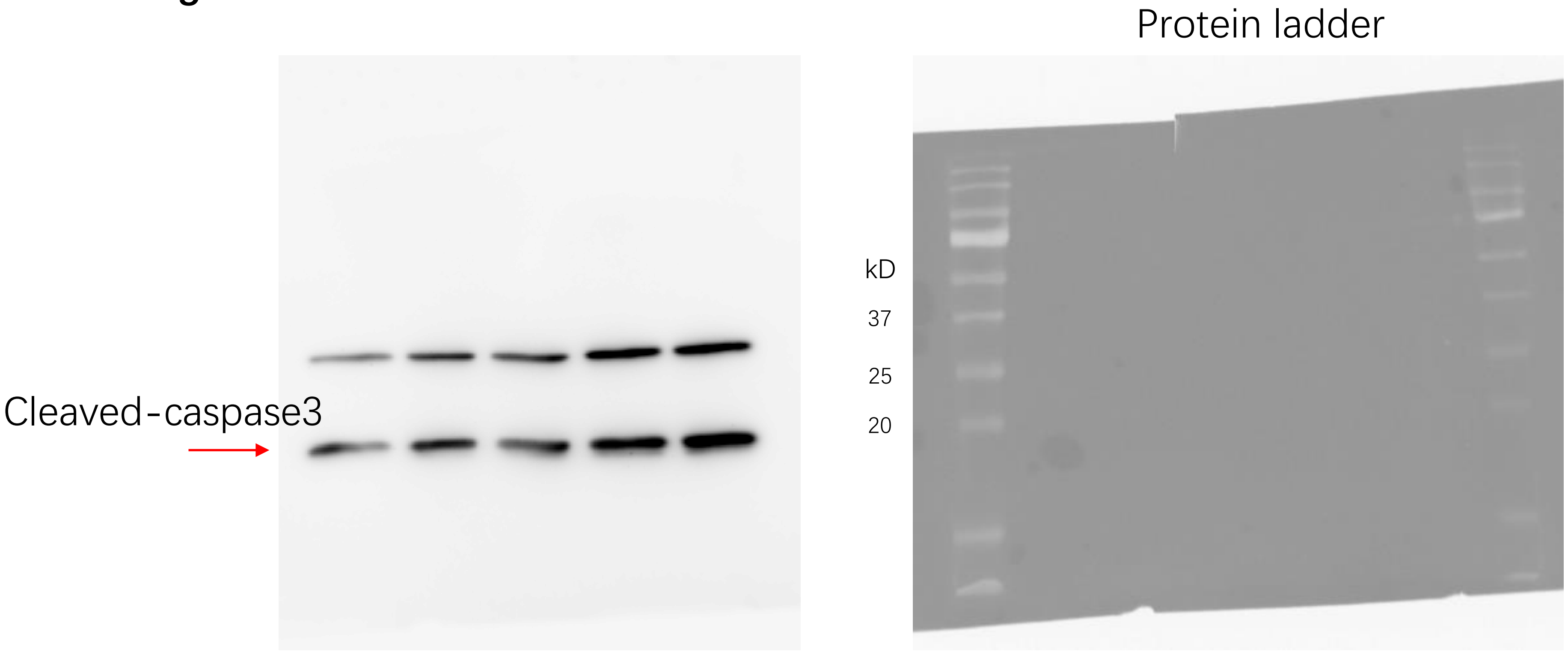

Figure 5C

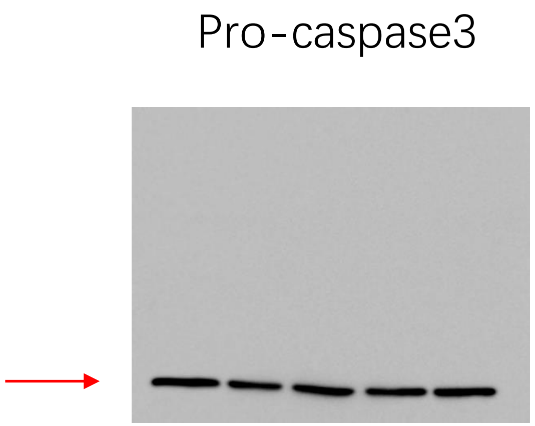

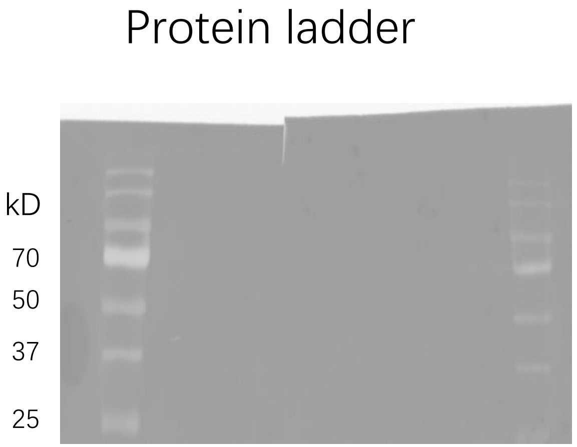

Figure 5C

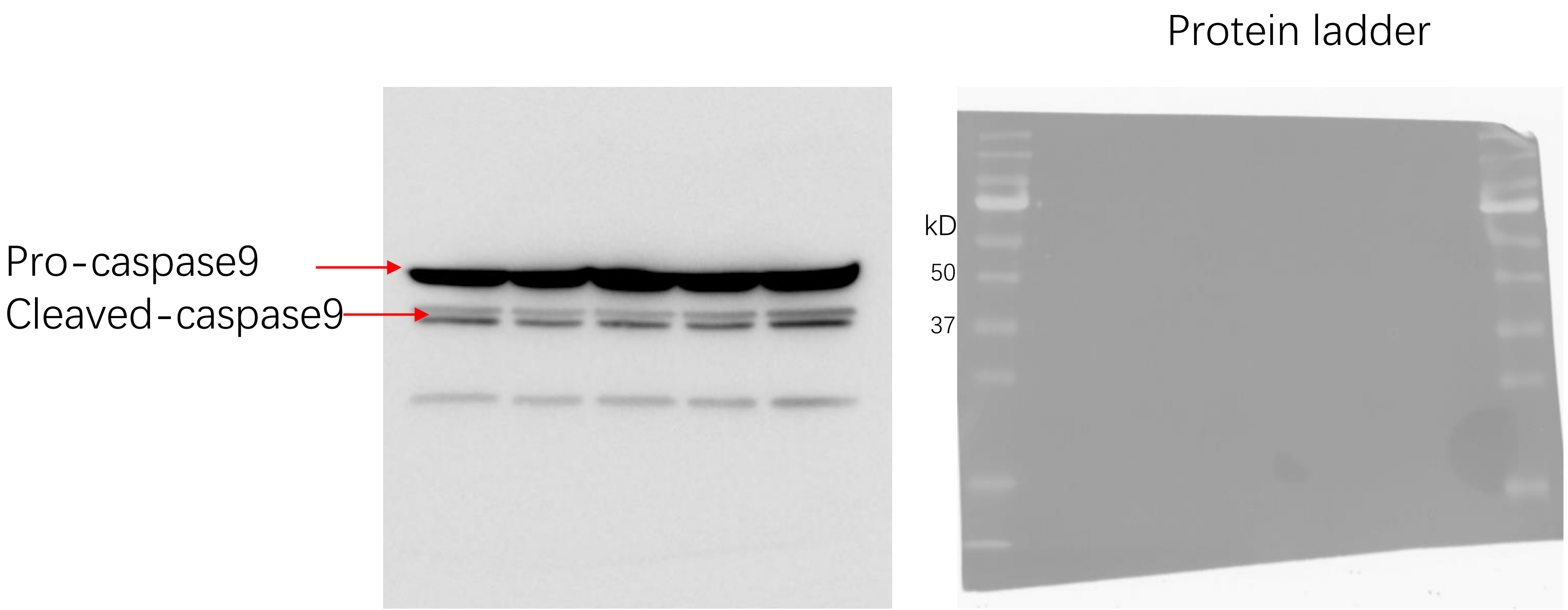

Figure 5C

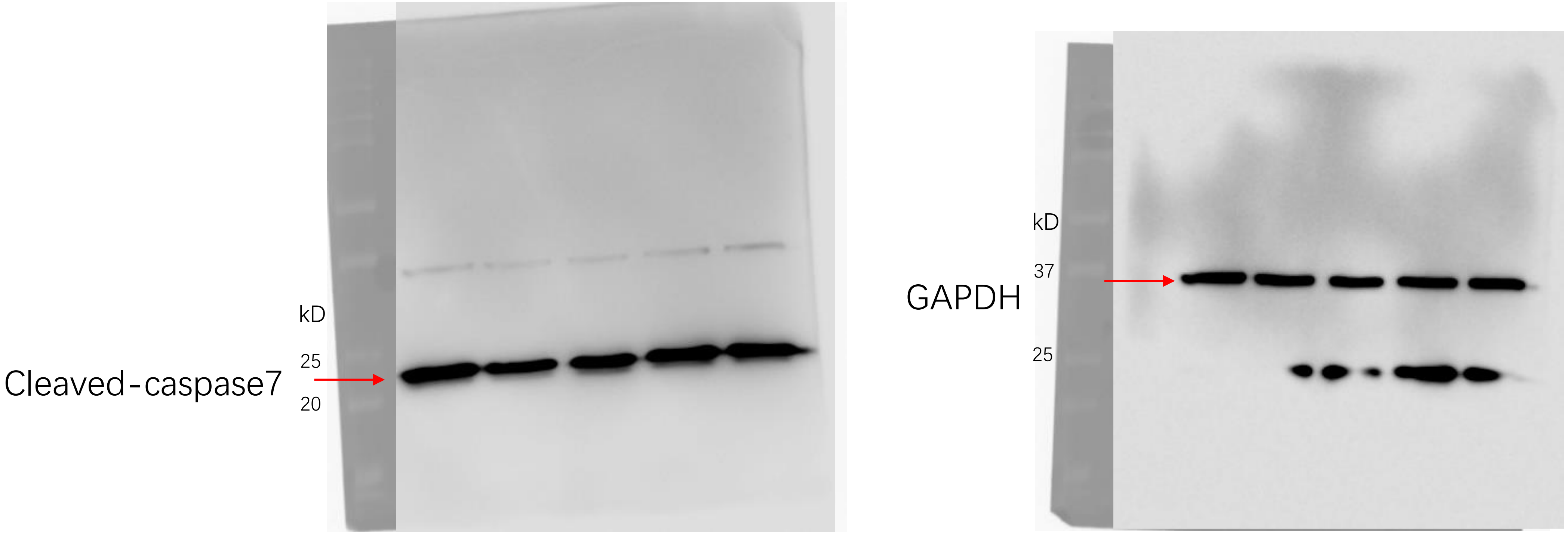

Supplement: Supplementary file 2 — Original Data File [file 41420_2023_1305_MOESM2_ESM.pdf]
